# Transcriptomic and Morphological Analysis of Cells Derived from Porcine Buccal Mucosa—Studies on an In Vitro Model

**DOI:** 10.3390/ani11010015

**Published:** 2020-12-24

**Authors:** Artur Bryja, Grzegorz Latosiński, Maurycy Jankowski, Ana Angelova Volponi, Paul Mozdziak, Jamil A. Shibli, Rut Bryl, Julia Spaczyńska, Hanna Piotrowska-Kempisty, Krzysztof Krawiec, Bartosz Kempisty, Marta Dyszkiewicz-Konwińska

**Affiliations:** 1 Department of Anatomy, Poznan University of Medical Sciences, 60-781 Poznań, Poland; abryja@ump.edu.pl (A.B.); mjankowski@ump.edu.pl (M.J.); rutbryl@gmail.com (R.B.); m.dyszkiewicz@ump.edu.pl (M.D.-K.); 2 Institute of Computing Science, Poznan University of Technology, 60-965 Poznań, Poland; grzegorz.lato2203@gmail.com (G.L.); krzysztof.krawiec@cs.put.poznan.pl (K.K.); 3 Department of Craniofacial Development and Stem Cell Biology, King’s College University of London, London WC2R 2LS, UK; ana.angelova@kcl.ac.uk; 4 Graduate Physiology Program, North Carolina State University, Raleigh, NC 27695, USA; pemozdzi@ncsu.edu; 5 Department of Periodontology and Oral Implantology, Dental Research Division, University of Guarulhos, Guarulhos 07030-010, SP, Brazil; jashibli@yahoo.com; 6 Department of Toxicology, Poznan University of Medical Sciences, 61-631 Poznań, Poland; julaspaczynska@gmail.com (J.S.); hpiotrow@ump.edu.pl (H.P.-K.); 7 Department of Histology and Embryology, Poznan University of Medical Sciences, 60-781 Poznań, Poland; 8 Department of Veterinary Surgery, Nicolaus Copernicus University in Torun, 87-100 Toruń, Poland; 9 Department of Biomaterials and Experimental Dentistry, Poznan University of Medical Sciences, 61-701 Poznań, Poland

**Keywords:** cell culture, oral mucosa, fibroblasts, DIC imaging

## Abstract

**Simple Summary:**

Domestic pigs express high phylogenetic similarity to humans and are often used as a compatible model in biomedical research. Porcine tissues are used as an accessible biomaterial in human skin transplants and tissue architecture reconstruction. We used transcriptional analysis to investigate the dynamics of complex biological system of the mucosa. Additionally, we performed computer analysis of microscopic images of cultured cells in vitro. Computer analysis of images identified epithelial cells and connective tissue cells in in vitro culture.

**Abstract:**

Transcriptional analysis and live-cell imaging are a powerful tool to investigate the dynamics of complex biological systems. In vitro expanded porcine oral mucosal cells, consisting of populations of epithelial and connective lineages, are interesting and complex systems for study via microarray transcriptomic assays to analyze gene expression profile. The transcriptomic analysis included 56 ontological groups with particular focus on 7 gene ontology groups that are related to the processes of differentiation and development. Most analyzed genes were upregulated after 7 days and downregulated after 15 and 30 days of in vitro culture. The performed transcriptomic analysis was then extended to include automated analysis of differential interference contrast microscopy (DIC) images obtained during in vitro culture. The analysis of DIC imaging allowed to identify the different populations of keratinocytes and fibroblasts during seven days of in vitro culture, and it was possible to evaluate the proportion of these two populations of cells. Porcine mucosa may be a suitable model for reference research on human tissues. In addition, it can provide a reference point for research on the use of cells, scaffolds, or tissues derived from transgenic animals for applications in human tissues reconstruction.

## 1. Introduction

Domestic pigs express high phylogenetic similarity to humans and are considered as compatible model in biomedical research [1,2]. Recently, advanced genetic engineering opened the possibility of genetically modified pigs’ application as tissue and/or organ donors in human xenotransplantation [3,4]. They were also used as a host organism for the formation of the heart and vascular architecture, to produce graft-ready artificial vessels [5,6,7]. Moreover, porcine tissues are an accessible biomaterial for human skin transplants and tissue architecture reconstruction [8,9]. Pigs are used worldwide, both as a model for biomedical research and an animal donor for tissue biopsies [10,11,12].

Our research focuses on the porcine oral mucosa, consisting of two distinct layers (epithelium and fibrous connective tissue layer), with remarkable capacity for repair [13,14,15]. The unique tissue architecture is intrinsically associated with cellular remodeling through cell survival and/or programmed death. Several morphological remodeling processes are essential for the repair process. In the case of cell/tissue primary in vitro culture, the modifications are also characterized by changes in transcriptomic, proteomic, and metabolomic profile [16,17,18,19]. In order to capture the regenerative capacity of the mucosa, we performed a microarray analysis of mucosa cells cultured in vitro. Moreover, it was shown that human fibroblast can be in vitro transformed to obtain cells with stem-like features [20,21,22], which is the basis for further translational research in humans and pigs.

Additionally, we analyzed microscopic images of cells cultured in vitro. Current approaches in cell imaging require many hours of manual adjustments and curation, sparking the need for development of new, supervised machine learning techniques. Live-cell imaging is a powerful tool in the investigation of complex biological systems at a single-cell resolution [23,24,25]. Nevertheless, the process of accurate image segmentation, needed to identify which parts of the image contain individual cells, often hampers the measurements [26]. The insight of single cell measurements provides us with information on the heterogeneous roles of the cells in a dynamic system and can shed light on constantly changing differences between populations. When imaging cells in tissues and in vitro conditions, it is important to obtain a high-quality image through differential interference contrast microscopy (DIC). Subsequently, the image can be processed via DIC image segmentation and reconstruction. DIC imaging offers a non-invasive visualization approach and has recently been developed in combination with retardation modulation [27,28]. Mathematical modeling and computer-assisted data analytics based on the imaging data collected from the in vitro samples is important to quantitatively assess stemness providing solutions and models to describe the biological processes, mechanisms, and their regulations on molecular and sub-molecular levels. Mathematical modelling and advanced image identification and machine learning are considered as attractive, novel methods, providing researchers with previously unknown insights. The objective of this study was to present the algorithmic pipeline, which may serve as a tool in assessing differentiation capabilities of cells in vitro.

Mathematical modeling of cellular data is an important new tool aimed at the task of understanding the biological mechanisms of cellular differentiation.

## 2. Materials and Methods

### 2.1. Animals

Thirty young gilts of the local Landrace, used in the experiment, were raised on a local commercial farm, and exhibited a mean age of 155 days (140–170) and weight of 100 kg (95–120). The housing and feeding conditions of the pigs were identical. All of the experiments performed in the study were approved by resolution 32/2012 (30.06.2012) of Local Animal Ethics Committee of the Poznan University of Life Sciences, Poland.

### 2.2. Buccal Mucosal Cells Isolation and In Vitro Culture

Samples of buccal mucosa were obtained within 40 min of slaughter and transported to the laboratory in the transportation medium (0.9% NaCl, 10 mg mL^−1^ streptomycin, 10 U mL^−1^ penicillin, 25 µg mL^−1^ amphotericin B). The excised tissue was washed twice in Dulbecco’s phosphate buffered saline (D-PBS; Merck, Darmstadt, Germany). The buccal mucosa was surgically removed using sterile surgical blades. The tissue fragments were minced and incubated with 1 mg mL^−1^ collagenase type I (Gibco, Life Technologies, Waltham, MA, USA) and 2 mg mL^−1^ dispase II (Gibco, Life Technologies, Waltham, MA, USA) for 2 h at 37 °C in a shaking water bath. The cell suspension obtained from this digestion was centrifuged at 300× *g* for 8 min. Supernatant was removed and pellet was resuspended in 0.25% trypsin solution (Merck, Darmstadt, Germany) for 10 min. Fetal bovine serum (FBS; Merck, Darmstadt, Germany) was used to neutralize trypsin. The cell suspension was filtered through mesh to remove non-dissociated tissue fragments, and then isolated cells were centrifugated at 300× *g* for 8 min. The final cell pellet was dissolved in Dulbecco’s modified Eagle’s medium (DMEM; Merck, Darmstadt, Germany) supplemented with 10% fetal bovine serum (FBS; Merck, Darmstadt, Germany) and 10 U mL^−1^ penicillin G, 10 mg mL−^1^ streptomycin, and 25 μg mL^−1^ amphotericin B (Antibiotic Antimycotic Solution; Merck, Darmstadt, Germany). Cell viability was 85% to 95% as determined using a cell counter Adam-MC (NanoEnTek, Seoul, Korea). The cells were maintained at 37 °C in a humidified atmosphere of 5% CO_2_. Once the cell cultures attained 70–80% confluency, they were passaged by washing with PBS (Merck, Darmstadt, Germany), digested with 0.25% trypsin solution (Merck, Darmstadt, Germany), neutralized using the same volume of FBS (Merck, Darmstadt, Germany), centrifuged (300× *g* for 8 min), and resuspended at a seeding density of 2 × 10^4^ cells cm^−2^. The culture medium was changed every three days.

In vitro primary cells culture was carried out for 30 days. During in vitro culture, daily photos were taken with the use of Olympus IX70 microscope (Olympus, Tokyo, Japan). In the periods of 7, 15, and 30 days, half of the cells were collected to isolate RNA and to perform microarray and real-time quantitative polymerase chain reaction (RT-qPCR) analysis.

### 2.3. Microarray Expression Analysis and Statistics

The in vitro cultured cells were collected and suspended in the TRI reagent (Merck, Darmstadt, Germany). The RNA isolation was based on the procedure described by Chomczynski and Sacchi [29]. Samples were collected at 7, 15, and 30 days of culture and subjected to double cDNA amplification (Ambion^®^ WT Expression Kit). The resulting cDNA was biotin labelled and fragmented using the Affymetrix GeneChip^®^ WT Terminal Labeling and Hybridization (Affymetrix, Life Technologies, Waltham, MA, USA). cDNA fragments prepared in that way (5.5 μg) were hybridized to the Affymetrix^®^ Porcine Gene 1.1 ST Array Strip (48 °C/20 h) (Affymetrix, Life Technologies, Waltham, MA, USA). The microarrays were then subjected to washing and staining based on the protocol of the Affymetrix GeneAtlas Fluidics Station. The scanning of the array strips was performed using the Imaging Station of the GeneAtlas System (Affymetrix, Life Technologies, Waltham, MA, USA). The following preliminary analysis was conducted with the use of the Affymetrix GeneAtlas^TM^ Operating Software (Affymetrix, Life Technologies, Waltham, MA, USA). The gene expression data quality was evaluated based on the quality control criteria included in the software. The resulting CEL files were imported into further software for downstream data analysis.

All of the graphs and analysis presented in the manuscript were compiled using Bioconductor 3.11 (Fred Hutchinson Cancer Research Center, Seattle, WA, USA) and R 3.6.3 programming language (R Foundation, Vienna, Austria). Each of the CEL files was merged with a corresponding description file. The Robust Multiarray Averaging (RMA) algorithm was used for result standardization, with moderated t-statistics of the empirical Bayes method performed to enable identification of statistically significant differences. Furthermore, Benjamini and Hochberg’s false discovery rate was used to correct the obtained p-value for multiple comparisons. The cutoff value for differentially expressed gene selection was assumed at *p* < 0.05 and expression fold change higher than |2|.

Differentially expressed genes were subjected to selection by examination of genes involved in cell migration regulation. The differentially expressed gene list (separated for up and downregulated genes) was uploaded to DAVID software (Database for Annotation, Visualization and Integrated Discovery) [30], where genes belonging to different gene ontology (GO) groups were obtained. The relation between those genes and selected GO terms were checked with GOplot R package [31]. The analysis included only those GO groups for which the P value was less than 0.05. The GO groups were selected to represent the processes occurring during in vitro culture. *Single–organism cellular process* (GO:0044763, *p* = 0.049155456) and *single–multicellular organism process* (GO:0044707, *p* = 0.000138853) represent processes that is carried out at the cellular level. *Regulation of cellular component organization* (GO:0051128, *p* = 0.000127787) includes processes involved in the formation or disassembly of cell structures. *Biological adhesion* (GO:0022610, *p* = 0.0000221), which includes the processes responsible for cell attachment to a substrate or another cell. Other processes responsible for the transition from proliferation to development and differentiation, *negative regulation of cell proliferation* (GO:0008285, *p* = 0.0000195), *developmental process* (GO:0032502, *p* = 0.000110534), and *anatomical structure development* (GO:0048856, *p* = 0.0000572).

String 10.0 software (Search Tool for the Retrieval of Interacting Genes; String Consortium, Zurich, Switzerland) was used for extraction of interactions between differentially expressed genes/proteins belonging to the analyzed gene ontology (GO) terms [32]. Interaction prediction query was based on a list of gene names. The search criteria were based on co-occurrences of genes/proteins in scientific texts (text mining), co-expression, and experimentally observed interactions. The results of such analyses generated a gene/protein interaction network where the intensity of the edges reflected the strength of the interaction score.

Finally, the REACTOME FIViz application, a part of the Cytoscape 3.7.2 software (Institute for Systems Biology, Seattle, WA, USA), was used to investigate the functional interactions between genes belonging to the chosen GO BP terms. The Reactome FIViz app (Reactome, Toronto, Canada) is used to examine pathways and network patterns related to cancer and other types of diseases. REACTOME accesses the pathways stored in the Reactome database, allowing to perform pathway enrichment analysis of a set of genes, visualize hit pathways using manually laid-out pathway diagrams directly in Cytoscape, and investigate functional relationships among genes in hit pathways. The application can also access the Reactome Functional Interaction (FI) network, a highly reliable, manually curated pathway-based protein functional interaction network covering over 60% of human proteins.

### 2.4. Real-Time Quantitative Polymerase Chain Reaction (RT-qPCR) Analysis

cDNA obtained during microarray analysis was used to validate the results using RT-qPCR technique. The RT-qPCR analysis was performed using LightCycler real-time PCR detection system (Roche Diagnostics GmbH, Mannheim, Germany), with SYBR^®^ Green I serving as a detection dye, and target cDNA quantified with the use of the relative quantification method. The relative abundance of *CCL8*, *CXCL2*, *DACH1*, *DUSP5*, *FABP5*, *IL6*, *PLK2*, *PPARD*, *PTGS2*, and *SPP1* was standardized to the internal standards ACTB and PBGD. For amplification, 2 µL of cDNA solution was added to 18 µl of QuantiTect^®^ SYBR^®^ Green PCR (Master Mix Qiagen GmbH, Hilden, Germany) and primers (Table 1). One RNA sample of each preparation was processed without the RT-reaction to provide a negative control for subsequent PCR. The average fold change in the gene expression of experimental samples were compared with control (7-day of in vitro culture) and calculated by 2^−ΔΔCt^ method. The results were presented as logarithm of fold change. Differences between experimental samples were estimated using the Kruskal–Wallis test with Tukey’s test. Statistical analysis was conducted using Excel with Realstats extension.

### 2.5. Automated Morphological Analysis of DIC Imaging

The mucosal cell cultures were visualized during their first 7 days of growth with differential interference contrast microscopy (DIC), using Olympus IX70 microscope (Olympus, Tokyo, Japan). After the microarray analysis, the morphological dynamics were investigated by analyzing the DIC images. The collected DIC images during in vitro culture were analyzed using a sequence of image processing and analysis operations, based on the collected data followed by quantitative assessment of selected relevant morphological features and their distributions. All methods described below were implemented using Python 3.5 (Python Software Foundation, Beaverton, OR, USA) and image processing libraries OpenCV 4.2.0 (OpenCV Foundation, Palo Alto, CA, USA) [33] and SciKit Image 0.16.1 (open source project; http://scikit-image.org/) [34].

#### 2.5.1. Image Processing and Segmentation

**Figure 1:**
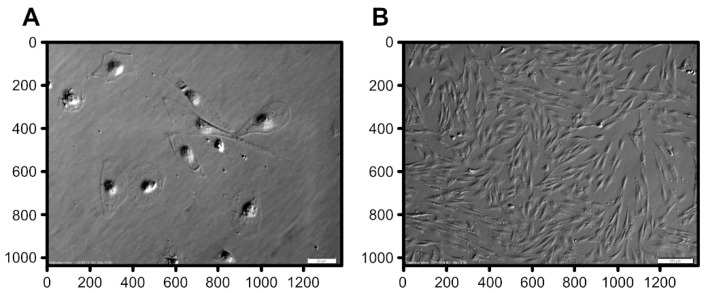
Raw DIC images considered in this study acquired at 40× (**A**) and 10× (**B**) magnification.

Representative images obtained during a 7-day in vitro culture were used to perform the analysis (Figure 1). The proposed algorithm starts from correcting the non-uniformities in scene lighting, which we achieve with low-pass filtering using Gaussian filter with a large mask. Subsequently, the crucial step of image segmentation follows, which is supposed to delineate well-defined *regions* (segments) from the input image. The interpretation of regions depends on the class of images with the goal to obtain exactly one separate region for each cell. It has been a historical challenge for one segment to accurately represent each individual cell using image analysis software if a cell is divided into two or more regions, it is referred to as *over-segmentation*; if two or more cells are merged in one region, this implies *under-segmentation*.

DIC images were analyzed using the *watershed segmentation* technique [35] and the OpenCV 4.2.0 software library (OpenCV Foundation, Palo Alto, CA, USA) [33]. The watershed algorithm builds regions in an iterative manner, where each region starts from a single pixel called a *seed* (a.k.a. *marker*). Feeding the algorithm with carefully selected seeds is thus essential for the quality of the outcome. Two methods for determining seed locations are either the Bespoke or the Feineigle method. With these two methods it was possible to determine seed locations. The Bespoke method first removes the pixels that are almost certain to belong to the background, with is achieved by normalizing image brightness, calculating image gradient using the Sobel filter, and then applying Otsu’s thresholding method [36]. The Feineigle method [37] attempts to reverse the physical process of image acquisition in DIC by directional integration of the input image. In brief, as a DIC image is essentially a gradient image of an unknown optical density function *f*(*x*,*y*) of the examined specimen, its integration should lead to the reconstruction of *f*, which is realized by formulating an optimization problem in a form of a set of equations that are subsequently solved using Gauss-Seidel relaxation.

In both above scenarios, the resulting segmentation undergoes further postprocessing. In particular, small artifacts (impurities) are removed, which we achieve by setting a magnification-specific minimum threshold of cell’s area (700, 1900, and 3000 pixels, respectively, for 10×, 20×, and 40× magnification).

The next stage of the analysis was the clustering process using k-means algorithm with the *k* parameter (the number of clusters) set to 3, which was applied to all 6080 cells gathered from all images. Additionally, the isomap dimensionality reduction technique was used to verify whether the obtained clusters are compact and thus adequately define classes of regions [38].

#### 2.5.2. Morphological Analysis

For morphological analysis and separation of keratinocyte and fibroblast populations of cells cultured in vitro, the following data were collected from each cell: Logarithm of the area, eccentricity, perimeter, major and minor axis length (the lengths of the axes of the ellipse that has the same normalized second central moments as the cell), and solidity (the ratio of the cell area and the area of its convex hull). To enable aggregation and comparison of measurements obtained from images of different resolution, we adopted the common measurement unit of 100 µm. There are 6 features that are calculated for each segmented cell individually (or more precisely for each region resulting from the above algorithms). For an image that contains n such regions, the features are gathered in an n × 6 array.

To capture the potentially multimodal characteristics of the cells in a given sample, we conduct cluster analysis on those arrays using a family of k-means algorithms.

### 2.6. Confocal Microscopic Observations of pCK Expression and Distribution

In 7, 15, and 30 days of in vitro culture, porcine buccal mucosal cells were collected and fixed in acetone-methanol solution (1:1) for 10 min at −20 °C. In the next steps, cells were washed, and to block non-specific binding, samples were incubated in 3% BSA with 0.1% Tween-20 for 30 min at room temperature (RT). Porcine oral mucosal cells were incubated at 1 h with a mouse monoclonal anti-cytokeratin 8 + 18 + 19 antibody (Abcam, GB). The antibodies were diluted 1:500 in PBS with 1.5% BSA and 0.1% Tween-20. After washing, the samples were incubated with MFP488-labeled goat anti-mouse IgG (MoBiTec GmbH, Göttingen, Germany) diluted 1:500 in PBS with 0.1% Tween-20 for 1 h at RT. A similar labeling process was carried out for fibroblasts isolated from porcine mucosa. Fibroblasts were used as a negative control. After incubation, samples were washed, and cells were suspended in 0.1 mL of mineral oil containing 1.5 μg mL^−1^ of 4′,6-diamidino-2-phenylindole (DAPI), mounted on an anti-fade medium on glass slide, and examined by an LSN 510 confocal system with an Olympus Fluoview 10i microscope (Olympus, Tokyo, Japan).

## 3. Results

### 3.1. Microarray Analysis

**Figure 2:**
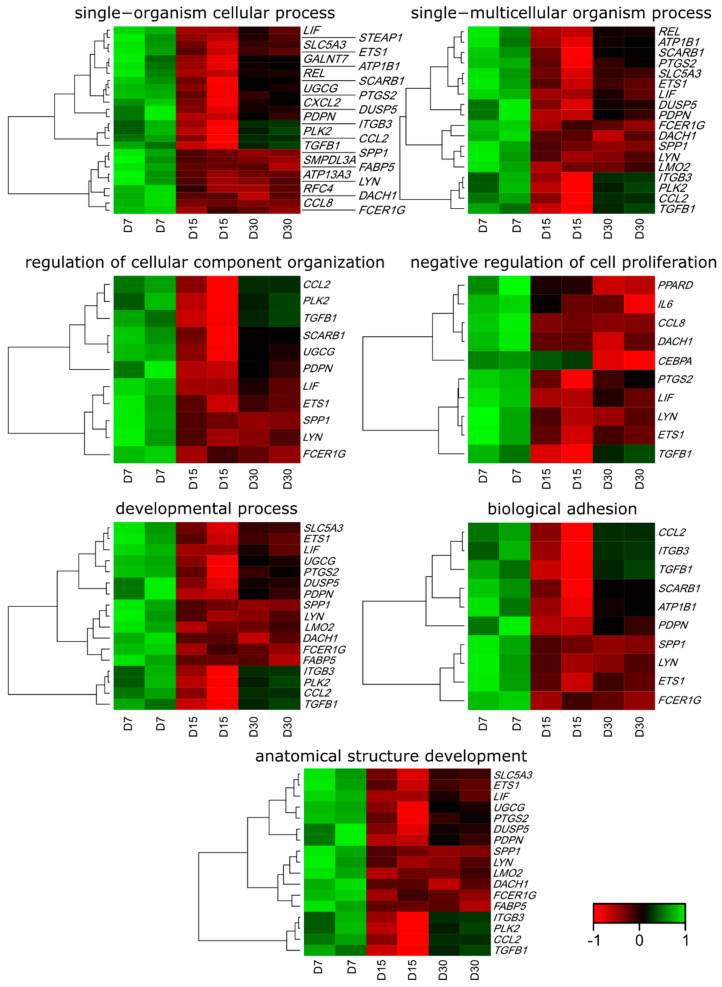
Heat map representation of differentially expressed genes belonging to the anatomical structure development, biological adhesion, developmental process, negative regulation of cell proliferation, regulation of cellular component organization, single−multicellular organism process, and single−organism cellular process gene ontology (GO) BP terms.

**Figure 3:**
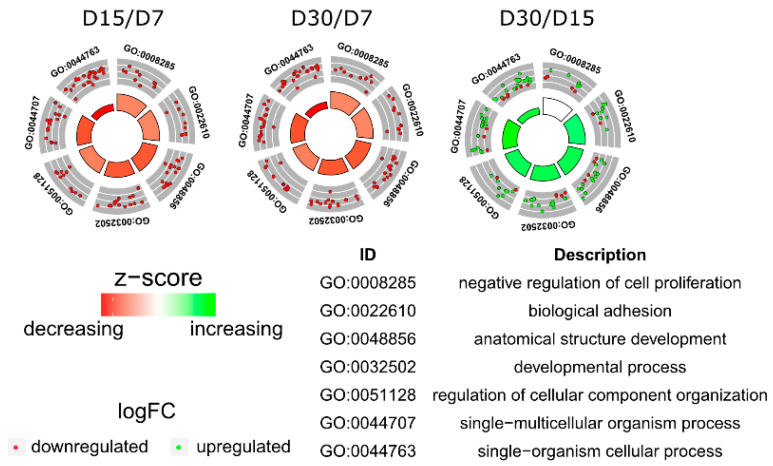
The circle plot showing the differently expressed genes and z-score of the anatomical structure development, biological adhesion, developmental process, negative regulation of cell proliferation, regulation of cellular component organization, single−multicellular organism process, and single−organism cellular process GO BP terms. The outer circle shows a scatter plot for each term of the fold change of the assigned genes. Green circles display upregulation and red ones downregulation. The inner circle shows the z-score of each GO BP term. The width of each bar corresponds to the number of genes within GO BP term and the color corresponds to the z-score.

**Figure 4:**
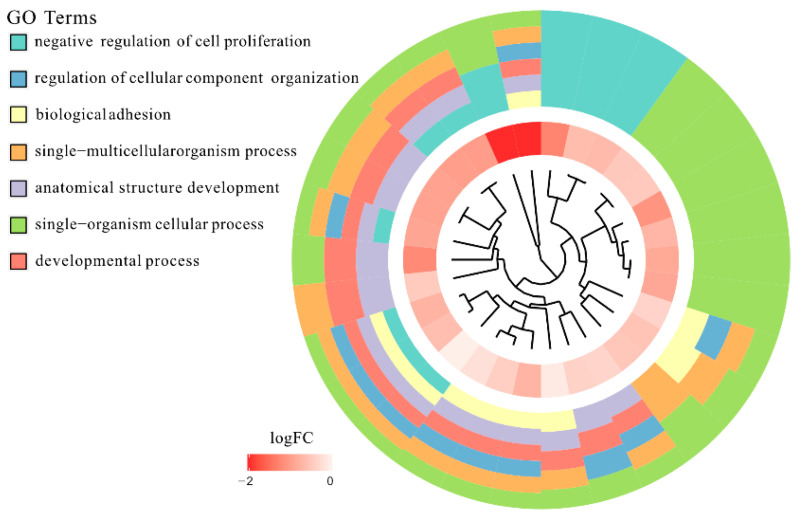
The representation of hierarchical clusterization, fold change, and assignment of differently expressed genes that belongs to chosen *anatomical structure development*, *biological adhesion*, *developmental process*, *negative regulation of cell proliferation*, *regulation of cellular component organization*, *single−multicellular organism process,* and *single−organism cellular process* GO BP terms. Genes are grouped together based on their expression patterns, and the clusterization pattern is represented by dendrogram inside the circle. The middle ring represents the logarithm of gene expression fold change of studied genes between 15 and 7 days of culture, respectively. The outer ring represents the terms assigned to the genes.

**Figure 5:**
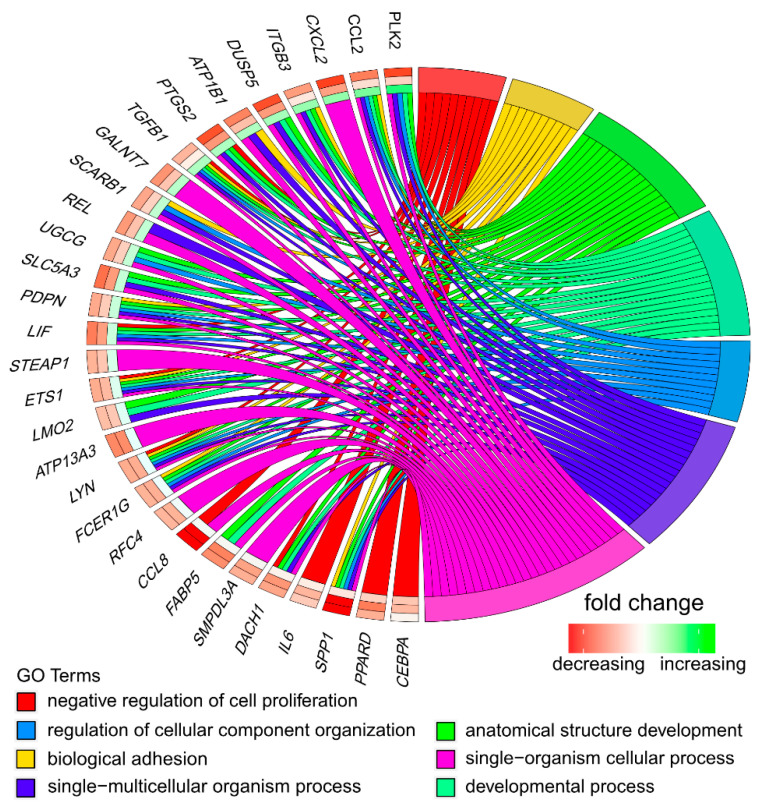
The representation of the mutual relationship of differently expressed genes that belongs to genes from anatomical structure development, biological adhesion, developmental process, negative regulation of cell proliferation, regulation of cellular component organization, single−multicellular organism process, and single−organism cellular process GO BP terms. The ribbons indicate which gene belongs to which categories. The middle circle represents logarithm from fold change (LogFC) between D15/D7, D30/D15, and D30/D15, respectively. The color of each block corresponds to the LogFC of each gene (green: Upregulated, red: Downregulated). The genes were sorted by logFC from most to least changed gene.

**Figure 6:**
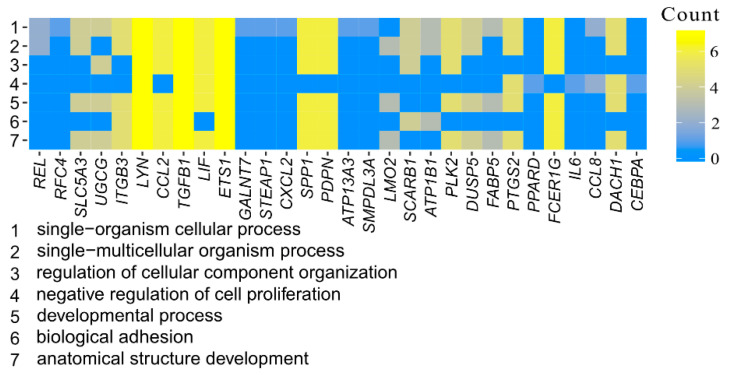
Heatmap showing the gene occurrence between differently expressed genes that belongs to anatomical structure development, biological adhesion, developmental process, negative regulation of cell proliferation, regulation of cellular component organization, single−multicellular organism process, and single−organism cellular process GO BP terms. The yellow color is associated with gene occurrence in the GO Term. The intensity of the color is corresponding to amount of GO BP terms that each gene belongs to.

**Figure 7:**
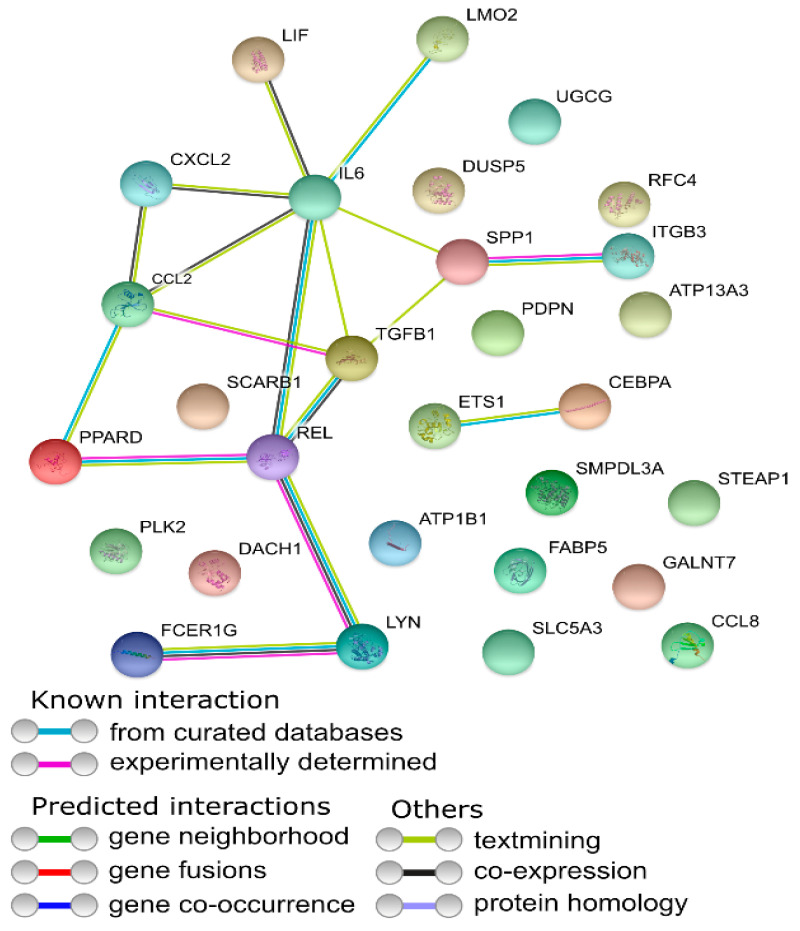
Search Tool for the Retrieval of Interacting Genes (STRING)-generated interaction occurrence between gene protein products that belongs to the anatomical structure development, biological adhesion, developmental process, negative regulation of cell proliferation, regulation of cellular component organization, single−multicellular organism process, and single−organism cellular process GO BP terms. Colored nodes reference to first shell of interactors, white nodes reference to second shell of interactors.

**Figure 8:**
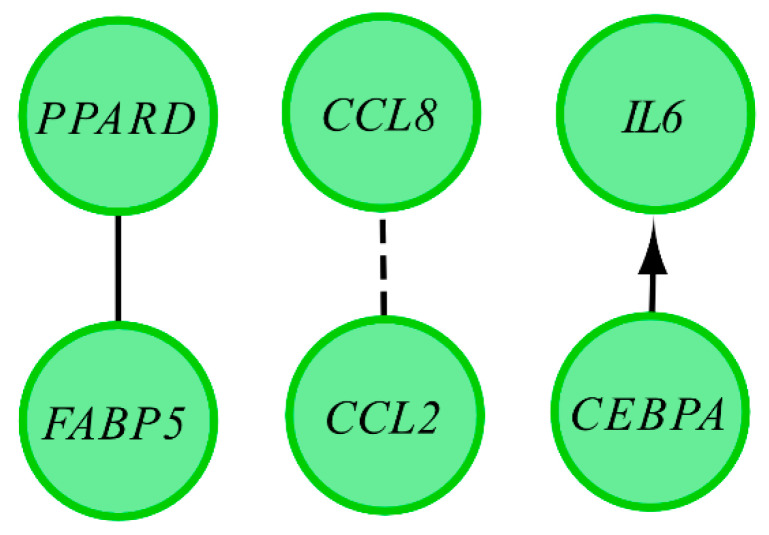
Functional interaction (FI) between 20 chosen differently expressed genes that belongs to the anatomical structure development, biological adhesion, developmental process, negative regulation of cell proliferation, regulation of cellular component organization, single−multicellular organism process, and single−organism cellular process GO BP terms. In the following figure, “->“ stands for activating/catalyzing, “-|” for inhibition, “-” for FIs extracted from complexes or inputs, and “---” for predicted FIs.

Affymetrix microarray profiling allows to analyze the gene expression changes between 7, 15, and 30 days of buccal pouch mucosa cells culture. The Affymetrix^®^ Porcine Gene 1.1 ST Array Strip was used to investigate the expression of 12,257 transcripts. Genes with fold change higher than abs (2) and with corrected *p* value lower than 0.05 were considered differentially expressed. This set of genes consisted of 130 different transcripts. Up and downregulated gene sets were uploaded to the Database for Annotation, Visualization and Integrated Discovery (DAVID) search separately, with only the gene sets with adjusted *p* value lower than 0.05 selected. The analysis showed that differently expressed genes can be classified to 56 gene ontology groups. This manuscript focuses on *anatomical structure development*, *biological adhesion*, *developmental process*, *negative regulation of cell proliferation*, *regulation of cellular component organization*, *single−multicellular organism process,* and *single−organism cellular process* GO BP terms. To present the genes as heatmaps the gene sets were subjected to hierarchical clusterization procedure and presented as heatmaps (Figure 2). On this basis, it can be concluded that comparing the results to the control level, which was day 7 of in vitro culture, the expression level of the analyzed genes was lower. For day 15, the exception is CCAAT enhancer binding protein alpha (*CEBPA*), for which the level of expression is similar to the control day. In the case of the 30th day of in vitro culture for the *CCL2*, *ITGB3*, *TGFB1*, and *PLK2* genes, the expression level is higher than on day 15 and is close to the control level. Numerical values corresponding to the data presented in heatmaps was presented in Table 2. The enrichment of each GO BP term between studied time periods were calculated as z-score and shown on the circle diagram (Figure 3). It has been confirmed that the expression for the analyzed genes was lower on days 15 and 30. In addition, an increased expression was found when comparing day 30 to day 15. Moreover, the genes can belong to multiple GO groups, making it important consider their overlap. The relation between those GO BP terms was presented as circular representation of clusterization (Figure 4), circle-plot (Figure 5), and as heatmap (Figure 6). In Figure 4, the outer ring shows the relationship between the analyzed ontological groups. The inner ring shows the level of gene expression on day 15 compared to day 7, confirming the reduction in expression level. Figure 5 shows that most of the analyzed genes belong to the *single−organism cellular process* GO BP term, and on the other hand, the groups *negative regulation of cell proliferation*, *biological adhesion,* and *regulation of cellular component organization* were represented by a smaller number of genes. Figure 6 shows that the three genes *LYN*, *TGFB1,* and *ETS1* were present in all analyzed ontological groups. Genes *SPP1*, *PDPN*, *FCER1G* were present in all ontological groups except *negative regulation of cell proliferation* GO BP term. LIF was presented in all GO groups except *biological adhesion* GO BP term. STRING interaction network was generated to visualize relations between the differentially expressed genes belonging to the selected GO BP terms. Using such prediction method provided us molecular interaction network formed between protein products of studied genes (Figure 7). Relationships between the protein products of the analyzed genes were detected for *REL* and *IL6*, which are the main centers for these relationships. The relationships for *REL* are stronger because they come from experiments, literature data, and databases. In the case of *IL6*, the interactions are mostly based on text data analysis. Finally, the functional interactions between chosen genes were evaluated with the REACTOME FIViz app to Cytoscape 3.7.2 software. The results are shown in Figure 8. The functional interactions were found for six genes, *PPARD* and *FABP5*, *CCL8* and *CCL2*, and *IL6* and *CEBPA*.

### 3.2. RT-qPCR Analysis

**Figure 9:**
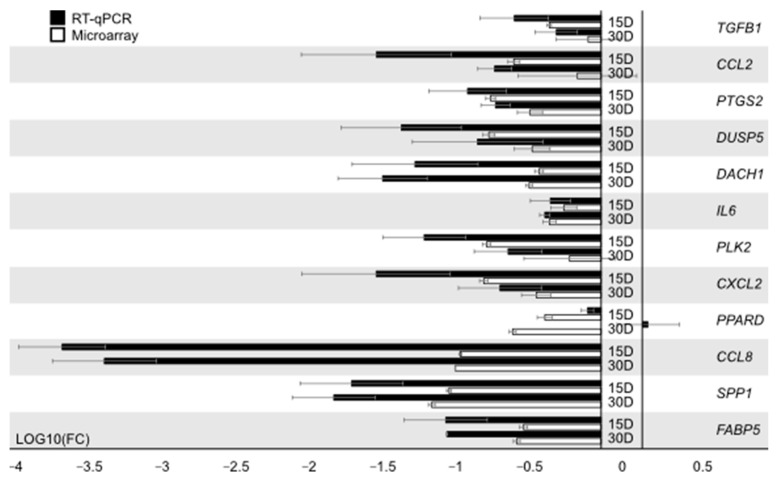
The results of RT-qPCR validation of the analyzed genes, presented in a form of bar graph.

Gene expression level in RT-qPCR was compared to the seventh day of in vitro culture. Direction and patterns of changes revealed by microarray analysis were confirmed via RT-qPCR (Figure 9). For one gene, *PPARD*, the direction of the change in expression was not confirmed. The variation in scale of changes could be noticed and easily explained by the fact that RT-qPCR is far more quantitatively accurate, as it focuses on a particular gene, as opposed to the full transcriptome. While microarrays are an excellent method of describing the patterns in gene expression, they need validation by other, more quantitative approaches. The differences may also be due to the fact that the method of measuring RNA amount for RT-qPCR and microarray is different.

### 3.3. Morphological Analysis

**Figure 10:**
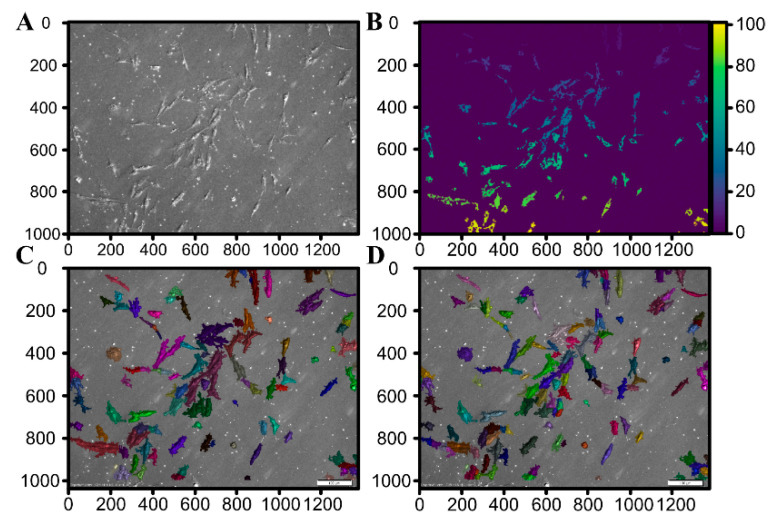
The process of image analysis for an exemplary image (**A**) (10× magnification). In (**B**), unique colors mark individual seeds for the segmentation algorithm. In the resulting images (**C**) and (**D**), colors mark separate regions created by the watershed segmentation algorithm.

**Figure 11:**
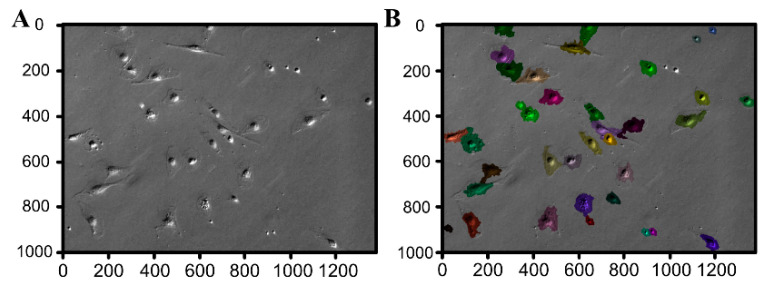
The outcome of the Feineigle-based method for an image (**A**) (40× magnifica-tion). In the resulting image (**B**), colors mark separate regions created by the watershed seg-mentation algorithm.

**Figure 12:**
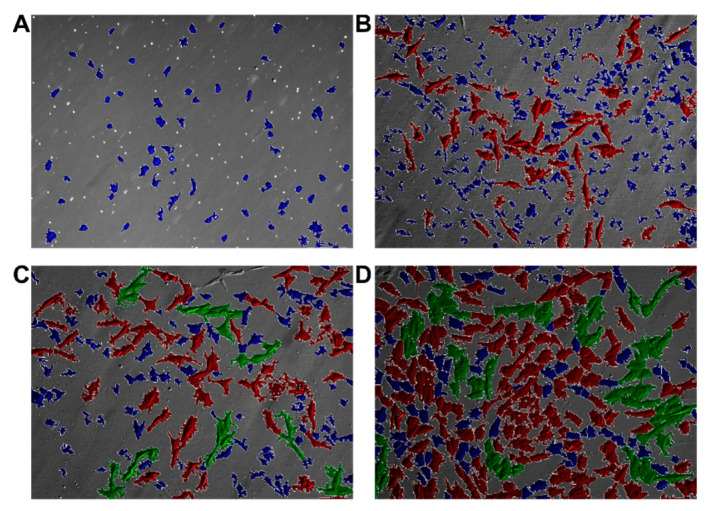
Segmentation outcomes for selected images acquired at, respectively, day 1 (**A**), 2 (**B**), 4 (**C**), and 7 (**D**) (10× resolution).

Based on the *watershed segmentation* and using the OpenCV software library, each connected group of pixels with brightness above the Otsu threshold was defined as a seed. Figure 10C presents the outcome of this process for an exemplary image in Figure 10A demonstrating that the Bespoke method proves quite reliable for small-magnification images (10× and 20×). Unfortunately, the Bespoke method leads to a relatively high degree of under-segmentation (notice for instance the large cluster of cells in the center of Figure 10C segmented as a single region marked in brown). To address the under-segmentation issue, images were post-processed by handling each region individually and performing local Otsu thresholding. The result is shown in Figure 10D; juxtaposing it with Figure 10C suggests that this post-processing significantly improves the outcome (the above-mentioned cluster of cells is now segmented into a few regions). For higher magnification (40×), the Bespoke method does not perform satisfactorily, making it necessary to employ the Feineigle technique. Once the Feineigle reconstruction is computed, it was subjected to two-threshold Otsu distance transform. The outcome of this process is illustrated in Figure 11. The outcomes of segmentation for several images acquired at several stages of culture growth are illustrated in Figure 12. Each colored patch identifies one region. Most regions delineate individual cells, though occasionally the segmentation algorithm groups a few cells together into one region, or misinterprets various image artefacts as cells.

**Figure 13:**
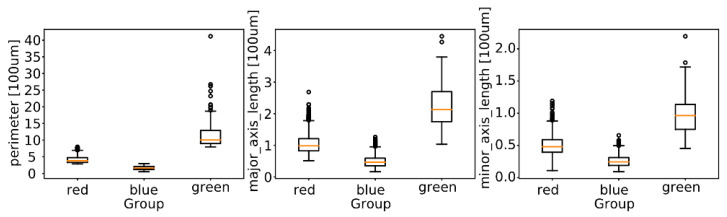
Distribution of most informative features across clusters: Perimeter, major axis, and minor axis. The boxes present median, first and third quartiles, and whiskers the furthest data point not further than from the quartile than 1.5 × IQR (interquartile range).

The colors of individual regions in Figure 12 reflect the outcome of the clustering process using k-means algorithm. The sizes of the clusters formed by the cells in our six-dimensional feature space are shown in Table 3. The two large clusters, referred to in the following as blue and red, form the main result of this analysis: Respectively, small, convex cells, and elongated, fiber-like cells. The smallest cluster groups mostly the regions in which the segmentation algorithm failed to delineate individual cells, and other strongly non-convex regions and artefacts, which are confirmed by Figure 13.

**Figure 14:**
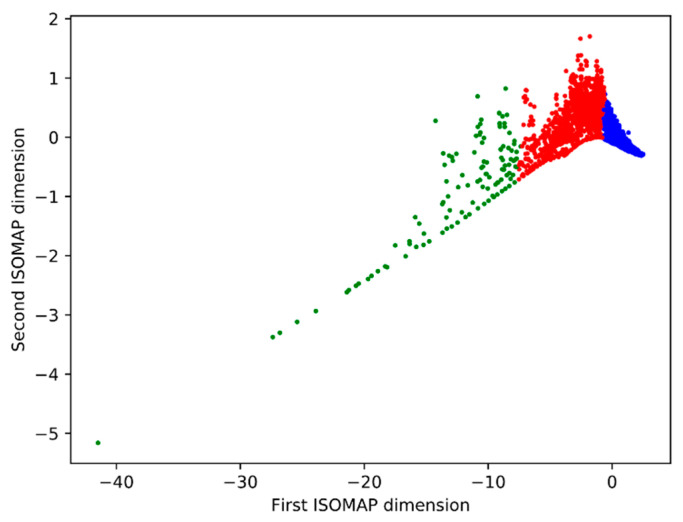
Projection of the six-dimensional feature data to two dimensions using the isomap algorithm Clusters defined by the clustering process, blue—keratinocyte-like cells, red—fibroblast-like cells, green—conglomerates of multiple cells.

An isomap dimensionality reduction technique showed that regions of type blue and red form a compact, dense cluster. This is not the for the regions of green type, given that those regions are mainly outliers, conglomerates of multiple cells (Figure 14).

**Figure 15:**
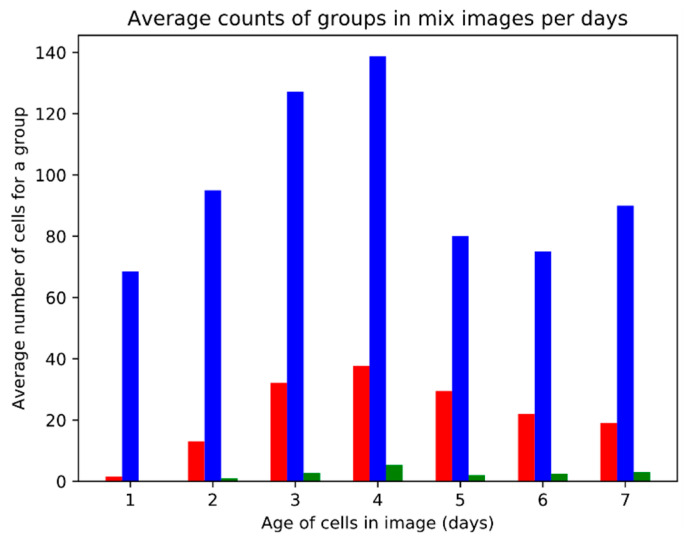
The average numbers of cells within the particular clusters (red, blue, green) as a function of the age of culture.

Figure 15 presents the timeline of cell types identified by the clustering process (for the mixed cultures), meant as the average number of regions per image. Interestingly, there is no clear monotonous trend: The numbers of both types of cells peak at day 4 and a relatively sudden drop on day 5 is observed. The proportion of red cells compared to blue ones starts from a very low value at day 1, increases roughly up to day 3, and then remains relatively stable. Outliers (the green group) are relatively rare across the entire timespan of experiment.

### 3.4. Confocal Microscope Observations of pCK Expression and Distribution

**Figure 16:**
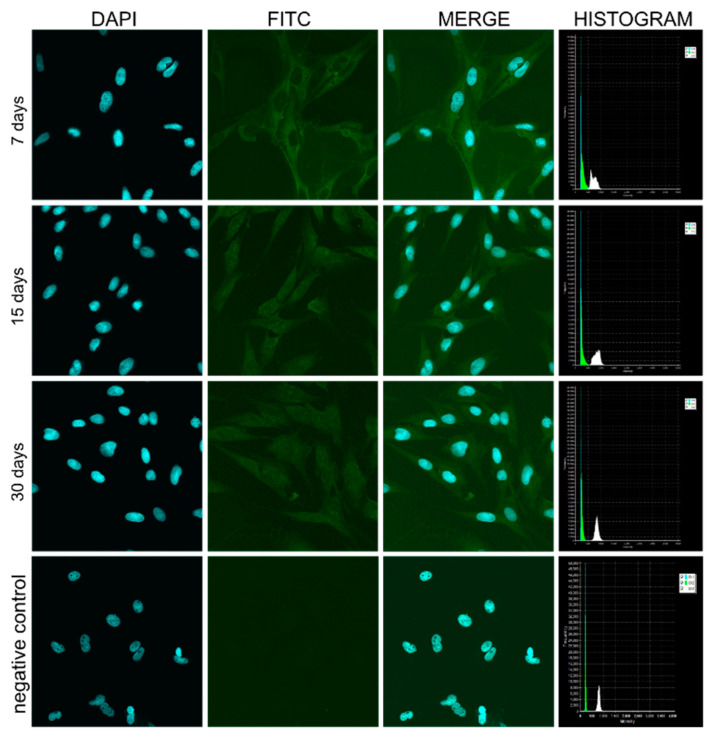
Confocal microscopic observation of cytokeratin 8 + 18 + 19 (pCK) levels and cellular distribution in keratinocytes in the designated periods of 7, 15, and 30 days of in vitro culture. pCK-labeled fibroblasts were used as a negative control. DAPI—4′,6-diamidino-2-phenylindole, FITC—fluorescein.

The automated analysis of DIC images showed majority of keratinocytes (76.33%) during the seven-day in vitro culture. To confirm the presence of keratinocytes in further days of in vitro culture, the distribution of cytokeratin 8 + 18 + 19 (pCK) in cells was detected using an immunohistochemistry and confocal microscope. The results obtained from this analysis are shown in Figure 16.

## 4. Discussion

Oral mucosa is an accessible tissue with remarkable capacity for repair. Composed of lining epithelium and supporting connective tissue, it allows to obtain two types of cells, keratinocytes and fibroblasts, in primary in vitro cultures. Interactions between these two different cell populations are important for the homeostasis of the tissue. Keratinocytes are described as small-sized (10–20 μm in diameter) polygonal cells that tend to change their morphology and size when undergoing differentiation process. During the differentiation process, they lose their ability to proliferate, and express irregular size and shape. In long-term in vitro conditions, these cells were reported to reach up to 50 μm in diameter [39].

The fibroblasts exhibit significantly diversified morphology, which depends on the location and different environmental conditions [40]. The tissue distribution of fibroblast subpopulations has a substantial impact on the regulation of connective tissue function [40]. These cells may show similar differentiation patterns as keratinocytes and similar expression patterns as mesenchymal cells [41]. One of the important processes for epithelial tissue, such is the lining of the oral mucosa, is the ability of wound healing, highlighting the importance of this tissue as a protective barrier towards the external and internal environment.

Analysis of primary cells obtained from porcine oral mucosa was performed using algorithms of DIC images, carried out on cell images over seven days of in vitro culture. The key step in image analytics of cellular cultures was the separation of individual cells from the background (and from each other, when two or more cells happen to be adjacent). The object separation stage, known as *image segmentation* in computer vision and pattern recognition, was particularly challenging, as the boundaries of cells render rather faintly in DIC imaging (Figure 1). Moreover, the DIC imaging process visualizes the cellular structures as a pseudo-3D relief, which, though convenient for humans, is not necessarily beneficial for image analysis algorithms. *Watershed segmentation* [35], with later improvements as implemented in the OpenCV software library [33], proved adequate and reliable in many past studies, and is routinely used also in biological and medical imaging. For example, OpenCV library was used to analyze images of skin cells, and quantitatively determine the percentage of damaged cells in the image [42]. Computer image analysis was also used when analyzing blood cells. The algorithm helped detect and count platelets, red, and white blood cells in blood samples [43]. Research was also conducted on the detection of malaria inpatients through microscopic digital image of blood sample and analysis using OpenCV library [44]. Jaccard et al. developed an algorithm for analysis of images acquired using phase contrast microscopy. Their algorithm allowed for automated image analysis and evaluation of key characteristics of cell cultures (confluency, morphology, and cell density) [45].

Some problems had to be solved during the computer analysis of DIC images. For higher magnification (40×), the Bespoke method does not perform satisfactorily, making it necessary to use the Feineigle technique. However, images at 10× magnification following the Bespoke segmentation method provided results that were visually more compelling than the Feineigle method for higher magnifications. Additionally, lower magnification allows the analysis of a larger numbers of cells and thus arrive at more significant statistical differences. Therefore, it is better to use images with a magnification of 10× for analysis.

Our algorithm applied a colored patch to each designated region. Most regions delineate individual cells, though occasionally the segmentation algorithm groups a few cells together into one region, or misinterprets various image artefacts as cells. However, the algorithm usually manages to identify individual cells even in the later stages of culture growth, when the density of cells is high and most of them are directly adjacent to other cells.

The algorithmic approach allowed the assessment of morphological types of cells that are present in the in vitro culture, providing the possibility for quantitative comparison between different morphological types of cells. Analysis of DIC images permits the determination of the percentage share of particular morphological types, which were followed by microarray analysis. As a result of the morphological assessment, two types of cells were selected. Some smaller and oval cells (blue color) that correspond to keratinocytes and second fiber-like cells (red color) that correspond to fibroblasts (Figure 4). The third type was a dispersed cluster (Figure 6) and corresponded rather to conglomerates of multiple cell types. Finally, after comparing the number of cells, oval cells accounted for the majority in the images analyzed, and fiber-like cells were the smaller group. This suggested the predominance of keratinocytes.

Following the evaluation of DIC images, a long-term culture in vitro was carried out. Long-term cultures are usually associated with a reduction in cells’ proliferative capacity and the transition to differentiation. This highlighted the importance to analyze the *negative regulation of cell proliferation* ontology group, associated with processes that reduce cell proliferation during our transcriptomic analysis. It is also important to analyze the ontological groups such as *regulation of cellular component organization* that consists of genes involved in formation or disassembly of cell structures, including the plasma membranes, and *developmental process* ontology group that consists of genes involved in the process of cell development and differentiation. We have also analyzed genes related to development, marked as *anatomical structure development* group. The other three groups, *single–organism cellular process*, *single–multicellular organism process,* and *biological adhesion* are related to cell adhesion and communication processes.

After comparing the obtained results, only three genes belonged to the ontological group containing genes responsible for stopping or reducing the proliferation process. The CCAAT/enhancer-binding protein alpha (*CEBPA*) interacts with CDK2 and CDK4 and reduces cell proliferation by inhibiting these kinases [46]. *CEBPA* can be expressed in basal keratinocytes. Increased co-expression of *CEBPA* and *CEBPB* was demonstrated in keratinocytes, that leave the basal layer and undergo terminal differentiation [47].

The *IL6* gene expression product is responsible for regulating the immune response process and haematopoiesis. *IL6* also influences the proliferation of normal and tumor-derived cells [48]. Previous studies showed that *IL6* inhibits proliferation and increases cell migration in the T47D breast carcinoma cell line [48]. Various cell types produce IL6. Among these cells are also keratinocytes and fibroblasts [49]. In human epidermal keratinocytes, IL6 can promote proliferation, but it is lower than proliferation induced by epidermal growth factor (EGF) or transforming growth factor alpha (TGFA) [49]. In another study, a sheet of mucosal cells was used to promote wound healing in skin. The authors observed early wound closure and limited scar tissue formation, with low-level expression of *IL6*, which is an inflammatory marker [50].

The *negative regulation of cell proliferation* ontology group included peroxisome proliferator-activated receptor delta (PPARD), which is a nuclear receptor that plays an important role in glucose and lipid homeostasis [51]. In the case of keratinocytes, it is a factor promoting the differentiation and accumulation of lipids [52,53]. It was also shown that PPARD activation in vivo did not display anti-proliferative or pro-apoptotic effects [52] and suggested that it inhibits the production of IL6 [54].

Three genes were upregulated after seven days of in vitro culture. After 15 days of in vitro culture, the expression of *CEBPA* was at a similar level with *IL6* and *PPARD* showing slightly lower level of expression. In the last period of long-term culture, the three genes described above were downregulated. Increased *CEBPA* expression after 7 and 15 days is consistent with previously reported results by Lopez et al. [47]. It appears that PPARD and IL6 could influence the differentiation processes of keratinocytes during in vitro culture.

*Single-organism cellular process* ontology group contains genes that influence processes carried out on cellular level. One of the genes present only in this ontological group is ATPase type 13A3 (*ATP13A3*), which is expressed in epithelial cells [17]. The high level of ATP13A3 may indicate the intensity of proliferation processes, and a significant decrease in its expression is associated with the process of apoptosis [17,55]. Another gene belonging to this ontological group is six transmembrane epithelial antigen of the prostate 1 (*STEAP1*) [17]. The protein product of this gene may play a role in intercellular communication. At the same time, the increase in *STEAP1* expression correlates with the increase in cell adhesion to the culture dish [17].

Among the analyzed ontological groups, only three genes were represented in all groups. Transforming growth factor beta 1 (*TGFB1*) was downregulated after 15 days of in vitro culture and was upregulated after 7 days and 30 days of in vitro culture [56]. TGFB1 is responsible for the control of cell proliferation and differentiation. It is secreted by fibroblasts and epithelial cells [56,57]. The next gene, v-ets erythroblastosis virus E26 oncogene homolog 1 (*ETS1*), regulates cell development. *ETS1* is normally expressed in the proliferative layer of epithelium. It was presented that ETS1 can block terminal differentiation of keratinocytes and promote their proliferation [58]. It was also showed that ETS1 induces expression of metalloproteases and might contribute to increased keratinocyte motility [58]. The last gene that belongs to all analyzed groups is v-yes-1 Yamaguchi sarcoma viral related oncogene homolog (*LYN*). The expression of *LYN* has been previously reported in the mouse keratinocytes [59,60]. It has been shown that LYN is a substrate for caspases, involved in the regulation of apoptosis and inflammation [60]. Phospholipase C gamma 1 (PLCG1) can be phosphorylated and activated by various tyrosine kinases, and one of these kinases is LYN [61]. Xie et al. showed that PLCG1 is required for calcium-induced keratinocytes differentiation, but in this process, mediated SRC and FYN kinases [61]. The next gene, sphingomyelin phosphodiesterase acid-like 3A (*SMPDL3A*), may be functionally related to other sphingomyelinases. It was investigated that *SMPDL3A* expression is regulated by liver X receptor (LXR) and may be species-specific [62]. It was also noticed no LXR-mediated induction of *SMPDL3A* expression in immortalized cell lines obtained from kidney, liver, and skin fibroblasts [62]. Otherwise, the diacylglycerol pathway, in which SMPDL3A is responsible for signal transduction, may be involved in regulating the differentiation of the epidermis [63]. Participation in the differentiation process of keratinocytes may also be controlled by polypeptide N-acetylgalactosaminyltransferase 7 (GALNT7), that can also regulate the osteogenic differentiation [64].

Replication factor C subunit 4 (*RFC4*) encodes a protein that is responsible for elongation DNA and was described in an earlier study [65]. Some data confirming the expression of *RFC4* in skin cells were presented by Phatak et al. [66]. RFC4 protein is involved in the post replication repair process and is expressed in proliferating cells.

Based on the analysis of the STRING interaction network and information contained in databases and comparison of text information from published scientific articles, there is a correlation between ETS1 and CEBPA. Since the increased expression of *CEBPA* induces the differentiation of keratinocytes, that migrating from the basal layer to the surface layers, we assumed that *ETS1* could also contribute to this process. ETS1, although it blocks the terminal differentiation of keratinocytes, may affect their mobility. Analysis of such dependence requires further studies. Most interactions were found for IL6 and v-rel reticuloendotheliosis viral oncogene homolog (REL), confirming the multi-regulatory function of IL6. A large number of interactions with REL generated by STRING is related to the fact that this factor belongs to the NFKB family. Lorenz et al. described REL as a key regulator of the growth and death of keratinocytes that may be playing an important role in epidermal carcinogenesis [67]. Another study showed that REL and RELA are necessary for normal epidermal development [68]. In the case of keratinocytes with silent *REL* expression, decreased proliferation was observed. These cells were very small and did not form colonies in culture [68].

Standardizing and normalizing primary cultures is essential to achieve repeatable results and to create a model that allows reliable analysis, which could bring us closer to translational research on oral mucosa regeneration.

## 5. Conclusions

The DIC images analysis algorithm that we used to extract cell images, and that further enabled us to map the different morphological populations among the cells obtained from porcine oral mucosa, is a great tool that could offer significant opportunities in future biomedical research. The use of machine learning allows to obtain more precise results, as well as to automate the entire process through the use of appropriate software. Automated image analysis can significantly reduce the costs associated with the identification of cell types and eliminate human errors. Further transcriptomic studies that followed the analysis obtained by DIC complemented our aim to characterize the cellular identities of oral mucosa in long-term in vitro conditions.

## Figures and Tables

**Table 1:** The sequences of RT-qPCR analysis primers.

Gene	Gene Accession Number	Primer Sequence (5′–3′)	Product Size (bp)
*CCL8*	NM_001164515.1	CAATGGAAAGATCCCCTTCA CTCGCAGTCCAGGTAGGAAG	206
*CXCL2*	NM_001001861.2	CTGTGACCAAACGGAAGTCA AGCCAAATGCATGAAACACA	237
*DACH1*	XM_001924267.6	GGCATGGACAACCACTACCT CTTTTGTTCCATCGCCAACT	233
*DUSP5*	XM_003359366	TGCACGACCCACCTACACTA GCGAGATCACACTCCTCCTC	250
*FABP5*	NM_001039746.2	ATGGCAAAGACCTCACCATC CGAGTGCAGGTGACATTGTT	244
*IL6*	NM_214399	TGTCGAGGCTGTGCAGATTA GCATTTGTGGTGGGGTTAGG	102
*PLK2*	XM_003133981	AGCCTGCTTCCAGACAAAAA GAAGGAGGTAGAGCCGAGGT	205
*PPARD*	NM_001130241	CAATGCCCTGGAACTCGATG TTGATCCGCTGCATCATCTG	249
*PTGS2*	NM_214321	AAAGGCCTCAATCGACCAGA ATCTGGGCGAGGCTTTTCTA	202
*SPP1*	NM_214023.1	ACTCCGATGAATCCGATGAG TCCGTCTCCTCACTTTCCAC	220

**Table 2:** Gene symbols, fold change in expression ratio, Entrez gene IDs, corrected *p* values, and mean value of fold change ratio of studied genes.

Gene	FC D7/D15	FC D7/D30	FC D15/D30	*p* Value D7/D15	*p* Value D7/D30	*p* Value D15/D30	Entez Gene ID
*SPP1*	0.0919949	0.0701564	0.762612	0.0162712	0.0230194	0.7663359	6696
*CCL8*	0.1098375	0.1015572	0.9246133	0.0099092	0.002855	0.8648287	6355
*CXCL2*	0.1585988	0.3618252	2.2813865	0.0282106	0.1000607	0.3277437	2920
*PLK2*	0.1660003	0.6091754	3.6697255	0.0282106	0.3101504	0.1884621	10769
*DUSP5*	0.1720052	0.3392398	1.9722648	0.0407438	0.1216767	0.4625691	1847
*PTGS2*	0.176579	0.3277974	1.8563786	0.0332722	0.0861828	0.447703	5743
*SLC5A3*	0.2252244	0.3389405	1.5049016	0.0282106	0.0555304	0.5402319	6526
*LIF*	0.252965	0.3537276	1.398326	0.0127508	0.0230194	0.3296551	3976
*CCL2*	0.2544943	0.6892844	2.7084476	0.0407438	0.4043909	0.2350893	6347
*ATP1B1*	0.2671037	0.4974017	1.8622043	0.0311185	0.1230303	0.3265278	481
*ATP13A3*	0.2795739	0.291783	1.0436704	0.0338446	0.0406862	0.9731139	79572
*FABP5*	0.2964051	0.2663389	0.8985637	0.0268357	0.0235015	0.8617697	2171
*GALNT7*	0.3118498	0.5252929	1.6844421	0.0410788	0.1507523	0.415765	51809
*REL*	0.33281	0.5087624	1.5286872	0.0234279	0.0563739	0.3173859	5966
*ITGB3*	0.3427886	0.7627618	2.2251672	0.038898	0.4213886	0.2350893	3690
*DACH1*	0.3777917	0.3230132	0.8550034	0.0298064	0.0235639	0.7580772	1602
*SCARB1*	0.3812346	0.5946187	1.5597186	0.0346365	0.1343271	0.3639803	949
*UGCG*	0.3838483	0.584988	1.5240083	0.0237712	0.0709872	0.283766	7357
*PDPN*	0.3878982	0.5660052	1.459159	0.0342294	0.1033121	0.4206462	10630
*LYN*	0.3967411	0.4074091	1.0268892	0.0346365	0.0423341	0.980695	4067
*ETS1*	0.3978662	0.4650034	1.1687432	0.0311185	0.0474721	0.7511418	2113
*SMPDL3A*	0.4101801	0.3641437	0.8877656	0.0300194	0.0244065	0.8019706	10924
*PPARD*	0.4137917	0.2496635	0.6033556	0.0520061	0.0235015	0.3504653	5467
*FCER1G*	0.4253226	0.4158717	0.9777794	0.0268357	0.0235654	0.9761984	2207
*STEAP1*	0.4256007	0.5300458	1.2454063	0.0268357	0.0442024	0.5192506	26872
*TGFB1*	0.443799	0.8154499	1.8374307	0.0150164	0.2166007	0.0719885	7040
*RFC4*	0.4463921	0.4260966	0.9545343	0.0346365	0.0362881	0.9445628	5984
*LMO2*	0.4833797	0.5386858	1.1144154	0.0268357	0.0348947	0.744229	4005
*IL6*	0.5575324	0.4445759	0.7973992	0.0884648	0.0442024	0.6294546	3569
*CEBPA*	0.8711062	0.4597589	0.5277875	0.3207823	0.017619	0.0452298	1050

**Table 3:** The sizes and proportions of clusters in the feature space.

Cluster	Number of Cells	Share
Blue	4641	76.33%
Red	1320	21.71%
Green	119	1.96%

## Data Availability

The data presented in this study are available in Gene Ex-pression Omnibus (GEO) database, accession number: GSE112659.
